# Primary dedifferentiated liposarcoma of the gallbladder: a case report and literature review

**DOI:** 10.3389/fsurg.2024.1452144

**Published:** 2024-11-13

**Authors:** Lan Wang, Tingting Lin, Yubin Hai, Kai Yu, Fan Bu, Ji Lu, Xiuli Wang, Miao Li, Xiaoju Shi

**Affiliations:** ^1^ Department of Hepatobiliary and Pancreatic Surgery, The First Hospital of Jilin University, Changchun, China; ^2^ Department of Urology, The First Hospital of Jilin University, Changchun, China; ^3^ Department of Plastic and Aesthetic Surgery, The First Hospital of Jilin University, Changchun, China; ^4^ Department of Pathology, The First Hospital of Jilin University, Changchun, Jilin, China

**Keywords:** treatment, dedifferentiated liposarcoma, diagnosis, gall bladder, case report

## Abstract

**Background:**

Liposarcoma (LPS) is a kind of malignancy of soft tissue usually found in the retroperitoneal, limb, or neck region, and some may be detected with delayed symptoms (pain or palpable mass), and less frequently occurs in organs of the digestive system. In contrast, Dedifferentiated liposarcoma (DDLPS) is a common histological subtype of LPS. The present study reported a case of dedifferentiated liposarcoma originating in the gallbladder. Differentiated liposarcoma originating from the gallbladder is rarely reported.

**Case description:**

A 64-year-old female patient presented to our hospital with a painless abdominal mass. Abdominal computed tomography (CT) showed that the gallbladder had lost its normal shape, and a 9.1 cm × 7.1 cm × 12.1 cm mass was seen in the area of the gallbladder fossa and the right upper abdomen below it, which had an irregular morphology, inhomogeneous density, and nodular calcification, with marked inhomogeneous enhancement on enhancement scan. Preoperative tumor markers and liver function indicators were not abnormal. With suspicion of a giant malignant tumor of the gallbladder, she underwent a cholecystectomy combined with abdominal mass resection. After surgery, the tumor and gallbladder, were completely resected, and postoperative pathological results confirmed the diagnosis of dedifferentiated liposarcoma deriving from gallbladder. After surgery, the patient and his family refused to continue treatment. After 15 months follow-up, the patient remains asymptomatic and does not show any signs of recurrence. And she is now under continued follow - up.

**Conclusions:**

Treatment of dedifferentiated liposarcoma is still at exploratory stage, and a lack of clinical evidence for this condition might hinder access to clinical trials and studies. Currently, the treatment of choice for dedifferentiated liposarcoma remains radical resection. In the available clinical studies, there are no robust data to support clinical use of neoadjuvant and adjuvant radiochemotherapy. As with other diseases, the use of radiotherapy and chemotherapy before and after surgery may be a potential future treatment.

## 1 Introduction

Liposarcoma (LPS) is the most frequent soft tissue sarcoma (STS) subtype in adults, accounting for 25% of all adult sarcomas (1–3). LPS is a malignant tumor of soft tissue usually found in the retroperitoneum, extremities, or neck region (4). It is divided into four main subtypes: (i) atypical liposarcoma (ALT)/highly differentiated liposarcoma (WDLPS); (ii) dedifferentiated liposarcoma (DDLPS); (iii) mucinous liposarcoma; and (iv) pleomorphic liposarcoma (5). Of these, WDLPS and DDLPS account for more than 60% of all LPS (6).

The term dedifferentiated liposarcoma, on the other hand, was first introduced by Evans in 1979 to define the morphological progression from atypical lipoma/highly differentiated liposarcoma to non-liposarcoma (7). DDLPS can be primary (90%) or can develop from preexisting ALT/WDLPS dedifferentiation (approximately 10%) (8). DDLPS is characterized histologically by progression from ALT/WDLPS to histologically graded sarcomas, with a shift from adipocyte-rich, well-differentiated areas of the tumor to non-adipogenic, spindle cell-rich areas (9). Macroscopically, DDLPS is a large, polymorphous yellow tumor with distinct non-lipomas (dedifferentiation) regions, which are solid and generally brown to grey (10). Despite the low metastatic potential of ALT/WDLPS, DDLPS is highly susceptible to distant pulmonary metastatic disease and locally recurrent (11, 12). The metastasis rate is 15%–20%, and the recurrence rate is nearly 50% (5, 13). There is very high local recurrence rate for DDLPS. DDLPS is refractory to chemotherapy and radiotherapy, and has a poor prognosis (14). Locally advanced DDLPS is incurable, and overall survival with palliative care is 11–20 months (15). Surgery is the main therapy for DDLPS because of the low response rate of DDLPS to conventional chemotherapeutic agents (16, 17). Complete resection is required to obtain a favourable outcome.

However, as the common locations of DDLPS are mostly retroperitoneal, limbs, and deep neck, the depth of the site makes it difficult to remove surgically, resulting in a local recurrence rate of nearly 50% and a poor prognosis (13, 18). The prevalence of DDLPS is less than 0.0001% annually and is therefore considered to be one of the rare cancers (19). Although DDLPS commonly develops in sites such as the retroperitoneum, some findings suggest an underestimation of the occurrence of nonperitoneal anatomical sites with this malignant disease (3). Case reports related to primary dedifferentiated liposarcoma of the digestive system are rare, and reports of primary dedifferentiated liposarcoma of the liver, gallbladder, and pancreas are even rarer. We report a case of gallbladder-derived dedifferentiated liposarcoma in a 64-year-old woman and review the available medical literature on primary dedifferentiated liposarcoma of the liver, gallbladder, and pancreas to summarize its epidemiology, etiology, clinical presentation, imaging features, diagnosis, and therapeutic options, including radiotherapy, chemotherapy, and targeted therapy.

## 2 Case report

### 2.1 History and examination

The patient, a 64-year-old woman, was found to have an abdominal mass on physical examination. One year before admission, the patient found a mass in her abdomen. Because of her poor financial situation, the patient was not admitted to the hospital. In the recent past, the patient perceived an increase in mass size. For seeking further treatment, she consulted our outpatient clinic. The patient reported no abdominal pain, no bloating, and no recent significant weight loss. The patient lives a regular lifestyle, has no history of smoking or alcohol consumption, and denies any history of exposure to toxic or radioactive substances. Her general medical history showed no operation or illness. Also, there was no history of any cancer in the family.

### 2.2 Imaging findings

**Figure 1:**
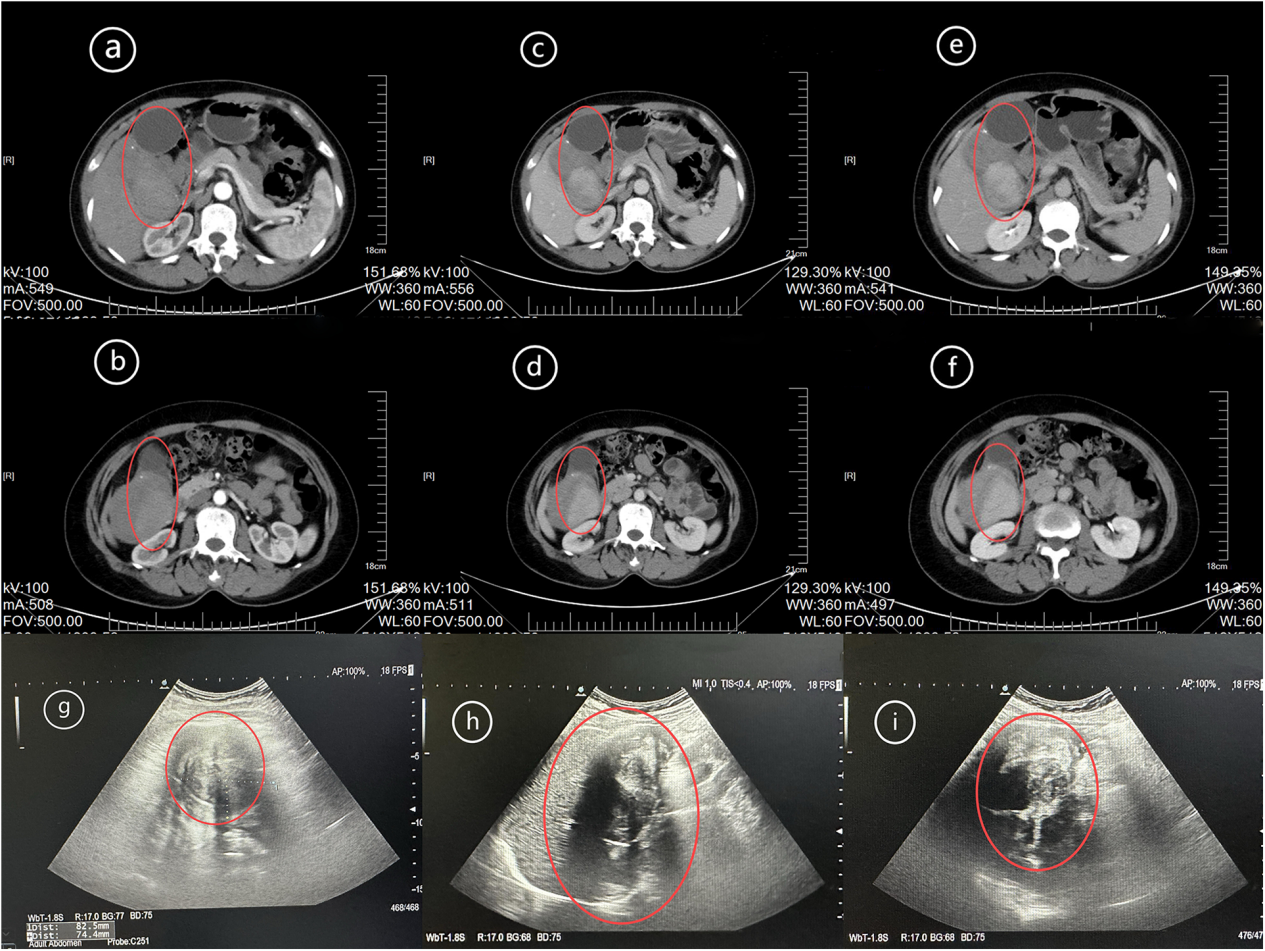
Radiological findings: the patient's dedifferentiated liposarcoma. Hepatobiliary, splenic, and pancreatic multi-row CT scan + three-phase enhancement: arterial phase **(a,b)**, portal phase **(c,d)**, and equilibrium phase **(e,f)** suggest an irregular large mass-like soft tissue shadow, with non-uniform density and a small amount of nodular calcification, and the enhancement scan shows obvious non-uniform enhancement (red circle); abdominal ultrasound **(g–i)** suggests a confined solid, strongly and weakly echogenic cluster, with non-uniform internal echogenicity, and irregular morphology (red circle).

The gallbladder had lost its normal shape, and an irregular large mass-like soft tissue shadow was seen in the area of the gallbladder fossa and the right upper abdomen below it, with inhomogeneous density and nodular calcification, and the enhancement scan was markedly inhomogeneous and strengthened, with a size of about 9.1 cm × 7.1 cm × 12.1 cm, which locally protruded into the neighboring liver, and was poorly demarcated from the liver and the duodenum as shown in Figure 1.

The patient also underwent a preoperative chest CT examination, which suggested a ground-glass nodule in the upper lobe of the right lung, measuring approximately 2.4 × 2.2 cm, and was considered to be a diagnosis of adenocarcinoma of the lungs, but did not visualise the mass in the gallbladder region. To further clarify the diagnosis of a mass in the gallbladder region, MRI should be performed, but the patient refused to undergo MRI in view of his financial condition and requested to receive surgical treatment as soon as possible.

### 2.3 Laboratory findings

The patient's tumor marker tests, liver function tests and the rest of the routine tests did not suggest significant abnormalities. Percutaneous biopsy is necessary because the diagnosis is not yet clear. However, the patient still refused to undergo puncture biopsy due to her financial condition.

### 2.4 Surgery

**Figure 2:**
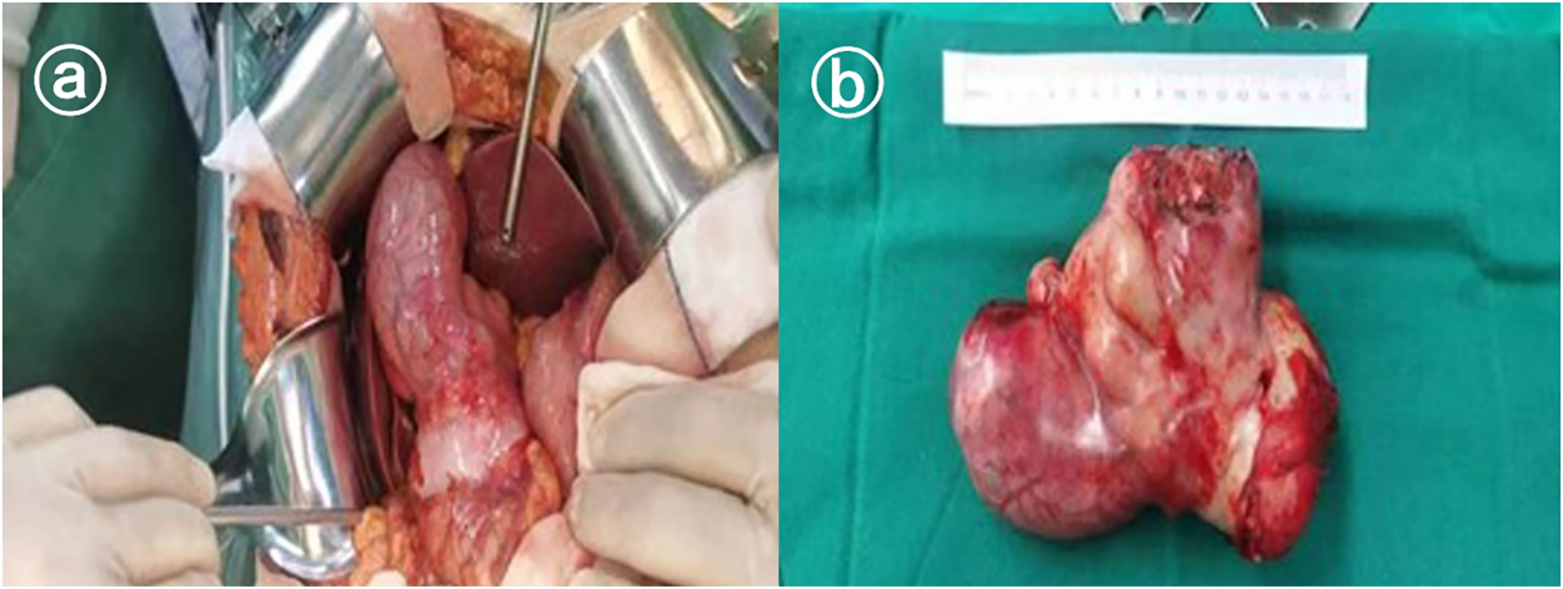
Gross observation of pleomorphic undifferentiated sarcoma. **(a)** Shows the intraoperative view and **(b)** shows the general view.

Preoperative imaging of the patient was more likely to be malignant, the tumor was poorly demarcated from the liver. Intraoperative observation of the general appearance of the tumor revealed that the tumor was irregular in shape, tough in texture, closely connected with the body of the gallbladder and the jugular abdomen, protruding into the abdominal cavity, adherent to the duodenum and the colon without invasion, and there were no obvious enlarged lymph nodes around the gallbladder and hepatic-duodenal ligament. The intraoperative rapid pathology return suggested that the tumor was of mesenchymal origin. To completely remove the tumor and minimize the chance of recurrence after surgery, the decision was made to surgically remove the gall bladder and tumor. The postoperative gross specimen showed that the tumor was solid and grayish-white, as shown in Figure 2.

### 2.5 Histopathological findings

**Figure 3:**
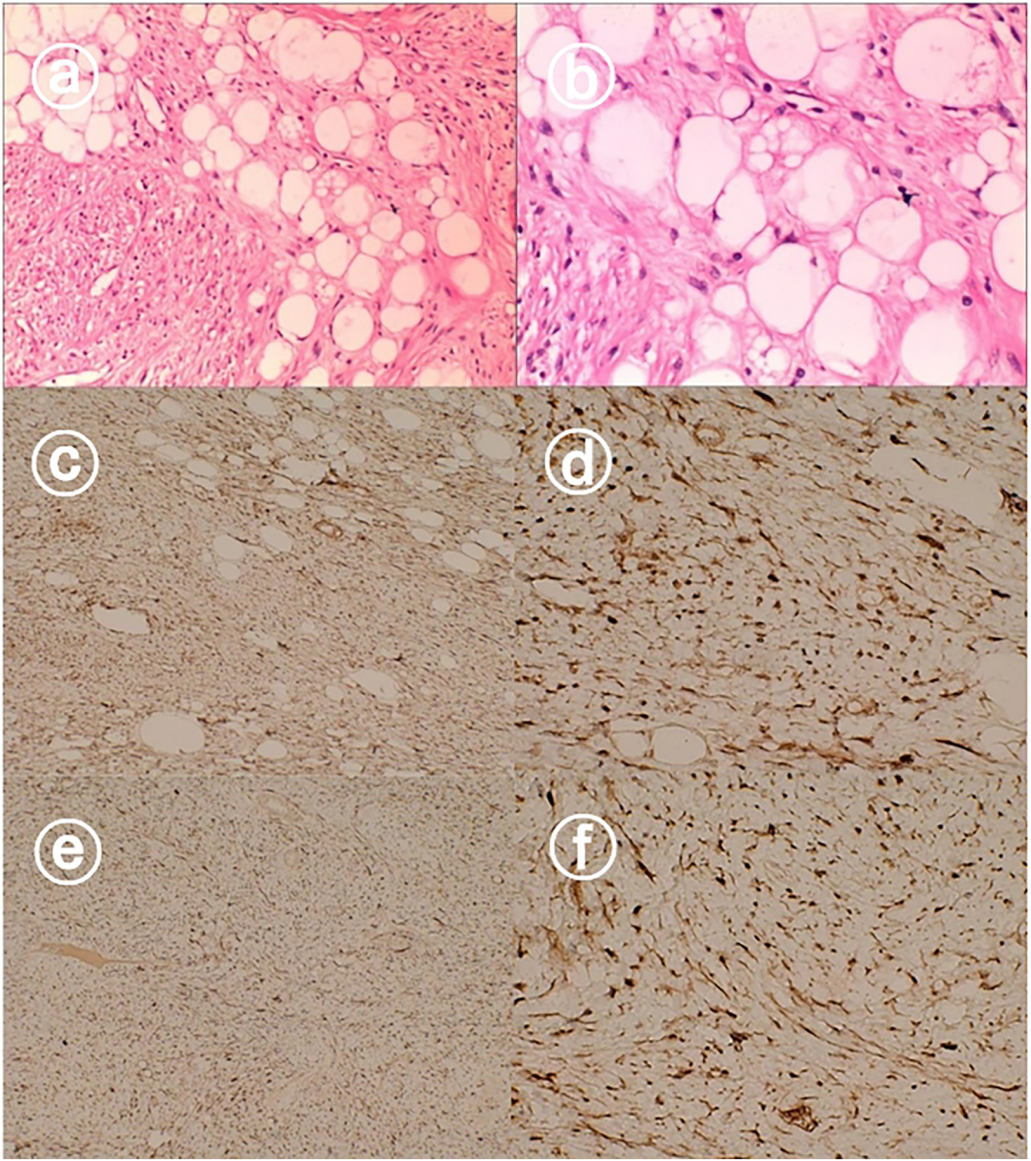
Hematoxylin and eosin staining showed dedifferentiated liposarcoma **(a,b)**. Immunohistochemical staining showed that the tumors were positive for CDK4 **(c,d)**, and MDM2 **(e,f)**.

Pathology showed dedifferentiated liposarcoma with a mass measuring 13 cm × 9 cm × 7 cm adjacent to the body of the gallbladder, grayish-white, solid, and tough on the cut surface. The gallbladder was 14 cm × 5 cm × 4 cm in size and pathologically showed chronic cholecystitis with no tumor seen at the cut edge of the cystic duct. The tumor was predominantly dedifferentiated component, the dedifferentiated component was low-grade sarcoma with an aggressive fibromatosis pattern and a small amount of heterogeneous component (bone tissue) was seen, the tumor involved the whole gallbladder wall, the size of the mass was 13 cm × 9 cm × 7 cm, there was no tumor infiltration of the vasculature and nerves. Immunohistochemistry: Ki-67(+8%), CD117(-), DOG-1(-), SDHB(+), CD34(-), SMA(focally +), S- 100(+), Desmin(focally +), β-catenin(membrane +), P16(+), MDM2(+), CDK4(+), CK4(+), MDM2(+). CDK4(+), CK-pan(-), ALK(-), HMB45(-). Abdominal malignancy, originating from the gallbladder, was consistent with dedifferentiated liposarcoma, and the associated images are shown in Figure 3.

### 2.6 Gene sequencing

The tumor mutation load (TMB) was 0.0 Muts/Mb, microsatellite stability (MSI) assay was microsatellite stable (MSS). Targets were detected: CDK4 amplification, copy number 16.0, and MDM2 amplification, copy number 28.0, with NCCN guidelines suggesting that 12q13-15 (CDK4, MDM2) amplification may be a molecular feature of dedifferentiated lipo sarcoma; CDK4 amplification, copy number 16.0, suggests potential sensitivity to Palbociclib (pipercetillin); MDM2 amplification, copy number 28.0, suggests sensitivity to Milademetan (DS-3032). No tumor-related genetic variants were identified.

### 2.7 Postoperative course

The patient had a good postoperative recovery with symptomatic treatment and was discharged after 7 days of surgical treatment without any immediate postoperative complications. Interestingly, the patient was found to have lung cancer in the same period, with a diameter of 1.8 cm and no local proliferation, and underwent radical surgery of wedge resection for lung cancer 1 month after surgery in our department, and the postoperative pathological return suggests non-mucinous infiltrative adenocarcinoma. At present, according to follow-up results, the patient's physical condition is good and there is no apparent abnormality. The specific follow-up process and results of the patients are as follows: The patient underwent abdominal ultrasonography in our hospital at 3 months, 9 months and 15 months postoperatively, which did not indicate tumor recurrence. For lung cancer, the patient also underwent chest CT examination at 2 and 8 months after lung surgery, which suggested no recurrence of lung cancer or lung metastases of dedifferentiated liposarcoma, except for a small amount of residual inflammation in the operative area.

## 3 Materials

Only eight cases have been reported, as shown in Table 1, of which three originated from the gallbladder (20–22). 4 cases originated from the pancreas (23–26) and 1 case originated from the liver (27). Table summarizes the 8 published cases of dedifferentiated liposarcoma of the liver, gallbladder, and pancreas. The median age of the patients was 68 years (range, 28–83 years), and all four patients older than 70 years presented with the dedifferentiated liposarcoma (DDLPS) subtype. Clinically, most patients presented with abdominal pain or no apparent discomfort [*n* = 5 (62.5%)], followed by a palpable mass [*n* = 3 (37.5%)], vomiting [*n* = 1 (12.5%)], fever [*n* = 2 (25%)], and dyspnea [*n* = 1 (12.5%)]. The most common site was the pancreas [*n* = 4 (50%)]. Tumor size varied from case to case, with a median size of 7.5 cm （range 4.1–28 cm). Follow-up information ranged from 2 to 48 months in 7 patients (87.5%) (mean: 17.7 months; median: 10 months), and 1 patient was lost to follow-up immediately after surgery. In the majority of cases where results were reported, the patients were alive. In contrast, one patient with primary dedifferentiated liposarcoma of the pancreas died of extensive systemic metastases 2 months after surgery.

**Table 1:** Review of dedifferentiated liposarcoma of the digestive system.

Author	Year	Age	Sex	Type	Size	Scope of involvement	Therapy	Prognosis
Adriano Carneiro da Costa	2018	71	Female	Dedifferentiated liposarcoma	14.2 × 9.5 × 13.8 cm	Gallbladder and part of the liver	A cholecystectomy associated resection with of Segments IV-B and V of the liver	Over 8 months
Rui-Qi Zou	2023	48	Female	Dedifferentiated liposarcoma	5.0 × 5.0 × 4.5 cm/7.0 × 6.0 × 5.0 cm	Gallbladder and part of the liver	Cholecystectomy with partial hepatectomy	Over 2 years
Cheng, Yung - Tsung	2020	83	Male	Dedifferentiated liposarcoma	3.2 × 4.3 × 6.0 cm	Gallbladder and part of the liver	Cholecystectomy with partial hepatectomy	Over 4 years
Yong Il Kim MD	1987	30	Female	The well - differentiated Liposarcoma and the cellular, non – lipogenic pleomorphic sarcoma	14 × 10 × 6 cm	The left lobe of liver	–	
Liu, Zhe	2019	28	Female	Well-differentiated Liposarcoma and dedifferentiated liposarcoma	28.0 cm × 19.0 cm × 8.0 cm	Distal pancreas	Distal pancreatectomy	Over 26 months
Tanabe, Masahiro	2022	81	Female	Dedifferentiated liposarcoma	75 mm	Distal pancreas and spleen	Distal Pancreatectomy with splenectomy	Over 55 days
Xiang, Han	2023	65	Female	Dedifferentiated liposarcoma	2.3 × 4.1 × 3.6 cm	Pancreas and spleen	Splenectomy and resection of the body and tail of pancreas	Died 2 months after surgery

## 4 Discussion

### 4.1 Epidemiology and etiology

The term “dedifferentiation” was first used in the medical literature in 1971, for progression from low-grade sarcomas to high-grade sarcomas, including low grade osteosarcomas, chondrosarcomas and fibrosarcomas. In 1979, the concept of dedifferentiation was further extended to include liposarcoma (LPS) (7, 28), i. e, dedifferentiated liposarcoma (DDLPS), which is characterized by a shift from adipocyte-rich, well - differentiated areas within the tumor to non-adipogenic, spindle cell-rich areas. Liposarcoma (LPS) is a major subtype of soft tissue sarcoma, representing 24% of the extremities and 45% of the retroperitoneal soft-tissue sarcomas (29). Four types of LPS have been identified. WDLPS and DDLPS are the most frequent, representing over 60% of total LPS (6, 30). DDLPS is a high grade invasion tumor, the incidence of metastatic disease is 5%–20% (29) and it also has a high tendency of local recurrence, which occurs in 40%–80% of patients even after major surgery (31). The presence of distant metastases, on the other hand, usually occurs concurrently with or after local recurrence (multivariate analysis: *p* = 0.0025). The local recurrence is related to a higher risk of distant metastatic disease [OR 4.46 (95% CI 1.67–13.40)] and thus leads to a poorer prognosis (32, 33). DDLPS is found in middle and elderly people, mostly from 50 to 70 (13). The majority of studies did not indicate a gender predisposition of DDLPS, however, in a recent trial involving 3,073 DDLPS patients from the U.S. National Cancer Data Bank, 65% were men (34).

The retroperitoneum is the most common location for DDLPS, which occurs very rarely in the extremities and subcutaneous tissue (5). Since DDLPS, which mainly affects the gallbladder, is extremely rare, as far as we know, there have been only three cases of this (20–22). All three patients presented with a painless mass on the right side of the abdomen, with or without fever and abdominal pain; tumor markers were not obviously abnormal after admission, except for one patient who had a high CA19-9 level of 49.50 U/ml (normal range, 0–37.0 U/ml); preoperative imaging in two of the patients suggested gallbladder stones; regarding the tumor, the gallbladder mass in all three patients had adhesion to the liver. liver with adhesions, and combined resection of the gallbladder, mass, and part of the liver was chosen for the operative procedure. Here, we report a rare case of gallbladder- induced DDLPS presenting as a 13 cm intra-abdominal mass in the gallbladder area.

### 4.2 Clinical presentation

DDLPS is usually discovered incidentally. Dedifferentiated liposarcoma patients usually do not have obvious specific symptoms. The clinical symptoms usually depend on tumor location and size. The patient in this case had no obvious abnormal signs and symptoms. In similar cases summarized in Table 1 and in the present case report, common symptoms included no obvious clinical symptoms in 3 cases (24, 25), abdominal mass in 3 cases (20, 21, 26), 3 cases of abdominal pain (22, 23, 27), 2 cases of fever (21, 23), emesis in 1 case (23) and dyspnea in 1 case (27). The following is a summary of the findings of the study.

### 4.3 Diagnosis and radiological characteristics

Due to its infrequency, imaging details regarding DDLPS are scarce in the literature. In general, because the imaging manifestations of dedifferentiated liposarcoma are nonspecific, regardless of the type of dedifferentiated liposarcoma component, in case of need for preoperative tissue diagnosis, biopsy should be performed on the components of the tumor in order to obtain accurate diagnosis.

Depending on the subtype, liposarcoma may display various radiological features, from purely fatty masses to non-specific soft-tissue masses that are invisible to the naked eye (35). Dedifferentiated liposarcoma presents in a variety of ways; however, the juxtaposition of non-liposarcoma areas within the fat mass and focal fat areas within the non-liposarcoma mass is a common pattern (36–38). DDLPS shares imaging features with WDLPS and is often suggestive of dedifferentiation by the presence of focal, nodular, and non-liposarcoma areas greater than 10 mm (39). These nonfatty lesions are easy to detect on MRI. Although WDLPS exhibits high signal intensity on both the T1WI and the T2WI sequences, the dedifferentiation area appears to display low-intensity regions on both sequences (40).

Imaging plays an important role in surgery. Generally, the operative edge should be selected 2–3 centimeters away from the lesion; as imaging technology advances, preoperative diagnosis of infiltration is increasingly consistent with postoperative pathology.

Although imaging cannot reliably distinguish between different types of liposarcoma, the existence of a definite non-liposarcoma mass in conjunction with a predominantly fatty tumor may indicate a dedifferentiated liposarcoma (38). In general, however, diagnostic imaging modalities are more limited in their ability to recognize disease. Pathology is the main gold standard for the diagnosis of this disease, with imaging generally being used as a reference and adjunct assessment. Care must be exercised to preserve the sample in pathological process and to prevent cross section, which might influence the evaluation of the depth and extent of invasion. For immunohistochemistry, cell cycle protein-dependent kinase 4 (CDK4) combined with human murine double minute 2 (MDM2) markers may be useful for diagnosing DDLPS. There is strong correlation between the expression of marker and the amplification of gene (41). In DDLPS patients, MDM2 and CDK4 genes were abnormally amplified on chromosome 12q14-15, and immunohistochemical staining suggested a high sensitivity for MDM2 and a high specificity for CDK4. Binh et al. reported that the diagnostic sensitivity of MDM2 immunopositivity was 95% with a specificity of 81% and that the diagnostic sensitivity of CDK4 immunopositivity was 92% with a specificity of 95%, thus, the combination of the two genes may be useful in diagnosing DDLPS (42). Therefore, the combined detection of these two genes has a characteristic diagnostic significance for DDLPS (43), and the higher amplification suggests a worse prognosis. Among the patients in this case report and the cases summarized in the above table, this patient presented typical abnormal amplification of MDM2 and CDK4 genes, while among the remaining 8 cases reported, 3 cases presented abnormal amplification of single MDM2 gene, 3 cases presented typical abnormal amplification of MDM2 and CDK4 genes, and 2 cases were not detected by fluorescence *in situ* hybridization. Compared with immunohistochemical fluorescence chemical staining, the detection of MDM2, CDK4 expression is a gold standard in diagnosis of DDLPS. Since rhabdomyosarcoma and peripheral malignant nerve sheath tumors, etc, can also abnormally express MDM2 and CDK4, and the sensitivity of p16 expression in DDLPS is 98%, combined detection of MDM2, CDK4, and p16 genes is often recommended to avoid misdiagnosis (44).

### 4.4 Treatment

For localized DDLPS, surgery remains the main therapy because DDLPS is largely resistant to conventional cytotoxic treatment (45). The patient in this case report and the remaining 8 patients underwent extended surgical resection, and except for a patient with primary DDLPS of the pancreas who died 2 months after undergoing extended surgical resection due to extensive systemic metastases, none of the other patients recurred during the follow-up period, and they achieved a relatively favorable prognosis. Extended operative excision for DDLPS increases overall survival (31). However, the local recurrence rate exceeds 80% despite aggressive surgery combined with systemic chemotherapy, the distant metastasis rate was as high as 20%, and the five-year disease-specific survival rate was 40%–60% (46). Systemic treatment is necessary for patients with locally advanced/unresectable, multi-recurrence or metastasis. It has been shown that systemic therapy combined with surgery reduces the chance of recurrence in patients with DDLPS, and neoadjuvant radiotherapy was associated with a reduction in the risk of locally recurrences in about one third of the patients (47). Currently, despite the promise of some targeted therapies, there is still a lack of effective well-tolerated treatments for DDLPS (48).

In clinical practices, standard local treatment of trunk and limb DDLPS includes wide resection with additional radiotherapy or amputation in the event of failure to save limb (49). Stage includes MRI and Chest Computed Tomography (CT) for the exclusion of lung metastatic disease. Patients with high grade LPS who have other risk factors (tumor size > 5 cm, depth of location in shallow fasciae or insufficient surgical margins) should receive neoadjuvant or adjuvant radiotherapy (50). Doxorubicin/ifosfamide is the standard regimen for patients selected for add-on chemotherapy. For small, high-grade tumors (<5 cm) that have been resected with good surgical margins, additional radiotherapy may not be necessary (13). In summary, the addition of adjuvant LPS to the treatment of small (>5 cm), high grade LPS and marginal resectable tumor may require consideration. Nevertheless, the choice of adjuvant treatment should be based on a multidisciplinary approach taking into account the specific drug susceptibility of each patient (51).

#### 4.4.1 Chemotherapy

Traditionally, systemic therapy options have been limited to cytotoxic chemotherapeutic agents like doxorubicin, isocyclophosphamide,gemcitabine, and docetaxel, that have been proven effective in unselected patients with soft tissue sarcoma (13). And there are now many systemic agents are available in patients with metastatic or unresectable DDLPS and WDLPS. Each patient's optimal treatment depends on a number of factors, including disease severity, physical condition, comorbidities, and symptoms of the patient.

The current standard systemic therapy for LPS consists of a doxorubicin monotherapy-based regimen as first-line treatment, with gemcitabine, docetaxel, trabectedin, and ezetimibe used for late-stage therapy (52). In a large-scale retrospective analysis, systemic therapy combined with chemotherapy had a limited role in STS subpopulation, with a remission rate of 12% (all with anthracycline-based chemotherapy), a median PFS (progression-free survival) of 4.6 months, and a median OS (overall survival) of 15.2 months (53). DDLPS has responded to chemotherapeutic agents and drug combinations, including doxorubicin (or doxorubicin in combination with isocyclophosphamide), gemcitabine (or gemcitabine in combination with docetaxel), trabectedin, eribulin, and pazopanib. However, several clinical studies have investigated the effectiveness of a combined combination of adriamycin and isocyclophosphamide vs. doxorubicin. They consistently demonstrated an improvement in disease response rates but no statistically significant difference in overall survival, at the cost of increased toxicity (54), these findings were recently confirmed in the EORTC 62012 phase III trial, which concluded that combination therapy significantly improved remission rates (26 vs. 14%, *p* < 0.0006) and median progression-free survival (7.4 months vs. 4.6 months, *p* = 0.003) (55). However, no significant benefit was observed in median overall survival (14.3 and 12.8 months, respectively, *p* = 0.073) as far as gemcitabine combined with docetaxel vs. gemcitabine monotherapy. There is no evidence yet of a difference in efficacy between the two, and for the late-stage therapeutic agents (gemcitabine-docetaxel,trabectedin, and eribulin), there are no randomized controlled trials that provide information on the sequence of therapy decisions; thus, selection of second line therapies is somewhat arbitrary and may be based on the comparative advantages of the various alternatives in the specific circumstances of each patient (55). However, DDLPS still has a high relapse rate with standard chemotherapy regimens, response rates are typically low, and response durations are typically short (52, 55).

Unfortunately, none of the eight patients enumerated in this article received postoperative treatment.

#### 4.4.2 Targeted therapy

Differentiated liposarcoma (DDLPS) is a kind of aggressive tumor with a poor prognosis. Low mutation load of tumor and frequent chromosome structural anomaly, including amplification of the chromosome 12q13-15 region and the MDM2 gene, are DDLPS's defining property (56). DDLPS is only moderately sensitive to radiotherapy and chemotherapy, and there is a clinical requirement for a more efficient therapeutic approach. The main therapeutic targets under investigation are the two overexpressed biomarkers of DDLPS, MDM2 and CDK4. MDM2 encodes an E3 ubiquitin-protein ligase, which binds to p53 to promote its proteasome-mediated degradation, thus negatively regulating its tumor suppressor function (57, 58). High and low MDM2 severely affects DDLPS cell proliferation, and high amplification levels of MDM2 is associated with poor outcomes in DDLPS (59, 60). What's interesting is that p53 is the MDM2 transcription factor that leads to a self-regulating feedback loop (61). Accordingly, restoration of p53 activity through the use of small-molecule inhibitors targeting the hydrophobic protein–protein interaction site between MDM2 and p53 has become a feasible targeted therapeutic strategy for various cancers (62, 63). The MDM2-p53 binding inhibitor (MDM2i) is active in DDLPS in preclinical DDLPS models, and it was demonstrated that it could recover the function of p53 (64). So far, multifold MDM2 antagonists have been studied (65). Nutlin- 3a is the first small molecule inhibitor that has been identified as a target for the p53 – MDM2 complex (66), displacing the p53 protein from MDM2 via its *cis*-imidazoline core structure. Shortly afterwards, improved MDM2- p53 complex inhibitors have been developed to increase specificity and efficacy (67). SAR405838, the non-Nutlin small molecular inhibitor MDM2, has also been studied in preclinical and clinical trials (68). HDM201, a newer inhibitor of MDM2, showed increased efficacy and selectivity (69). However, there is little prospect of efficacy with MDM2 inhibitors alone in therapy (70), MDM2 suppression was linked to elevated p53 protein expression in pre-clinical and clinical studies, but it was not linked to an improvement in the results of tumour growth suppression or prolonged survival (71). Cyclin-Dependent kinase-4 (CDK4) plays a key role in the transformation of G1 cell cycle. CDK4 binds to D-type cell cycle proteins (CyclinDI-D3) and phosphorylates Rb family proteins (Rb, p107, p130) to initiate the early G1 cell cycle transition (72), CDK4/6 phosphorylates the retinoblastoma tumor-suppressor protein (Rb1), which in turn dissociates Rb1 from the transcription factor E2F and allows progression of the cell cycle from the G1 to S phases. Clinical studies looking at palbociclib CDK4/6 inhibitors have shown modest clinical benefit. Dickson et al. conducted a phase II, non-randomized clinical trial in which patients with advanced WDLPS and DDLPS received palbociclib. Of the 28 patients, 57% achieved progression-free survival at 12 weeks and one patient achieved durable complete response 2 years after treatment (73), the results are still not satisfactory. Because of the unsatisfactory outcome of single therapy with CDK4/6 inhibitors, CDK4/6 combined with MDM2 is becoming more and more conspicuous. In the Phase Ib trial in patients with advanced WDLPS or DDLPS, combining an MDM2 inhibitor with a CDK4/6 inhibitor ribociclib was demonstrated to have an initial anti-tumor effect and a controlled safety profile (74). Telomeres may serve as potential therapeutic targets of DDLPS. Telomere maintenance plays an important role in ensuring the longevity of cancer cells. In a study by Irene Alessandrini et al, the impact of RHPS4, a molecule capable of altering telomeres by binding to telomere structures known as G-quadruplexes in patient-derived DDLPS cell lines, was evaluated, and the results suggest that RHPS4 could serve as a potential therapeutic approach for DDLPS (75). AXL, a well-characterized tumor promotion receptor tyrosine kinases with high levels of expression and activation in many types of tumors and sarcoma, including an aggressive subtype of liposarcoma, was also evaluated in a study by May, Caitlin D et al. The results indicated that AXL is responsible for DDLPS and PLS's aggression, so AXL may be a promising candidate in treating this kind of rare but destructive cancer (76). There are also a number of targeted drugs that are being investigated, such as Exportin 1 Inhibitors and PPARγ Agonists and so on. Exportin 1 (XPO1) plays an important role in the nuclear export of over 200 proteins, many of which are tumor suppressor. Overexpression of XPO1 has been observed in a variety of cancers including LPS (77). PPARγ is a kind of nuclear receptor that regulates the expression of certain genes essential to the development of fat cells in the end. LPS cells are induced to differentiate *in vitro* on PPARγ agonist exposure (78). However, clinical trials on specific drugs are still underway (51).

Overall, most of the DDLPS targeted treatment trials have shown poor clinical efficacy when compared with the objective response rate estimated to be 26% for DDLPS first-line anthracycline-based chemotherapy (55). For this reason, most of the targeted inhibitors under investigation as add-on to systemic treatment are not recommended at this time. As for the remaining potential therapeutic targets, further in-depth studies are needed.

#### 4.4.3 Radiotherapy

Regarding the efficacy of radiotherapy, most researchers believe that local radiotherapy is more efficient for uninfiltrated tumors (79).

However, there is no clear evidence that localized radiotherapy is beneficial for DDLPS. One study showed that in patients with retroperitoneal liposarcoma, neoadjuvant radiotherapy was not associated with OS for PS - matched DDLPS (HR 1.02, *p* = 0.889) (80). A propensity-matched analysis of 3,911 patients with primary limited retroperitoneal liposarcoma from the U.S. National Cancer Database (2004–2017) showed that radiation therapy only affected OS, not DFS (80). There were 2,252 patients with WDLPS, and 1,659 patients had DDLPS. After a median follow-up of 4.1 years, the median OS was 10.7 years. After propensity score matching, neither postoperative nor preoperative addition of radiotherapy significantly improved OS in the liposarcoma group. However, in meta-analyses, patients with highly differentiated liposarcoma had a tendency to improve overall survival after preoperative radiotherapy (HR = 1.80, 95% CI 0.95–3.42, *p* = 0.067). In the pooled cohort analysis, preoperative radiotherapy was associated with improved 5-year abdominal recurrence-free survival in patients with high grade liposarcoma and grade 1 or 2 dedifferentiated liposarcoma (*n* = 266, 65.8% vs. 56.0%, HR = 0.63, 95% CI 0.40–0.97), but not in patients with grade 3 dedifferentiated liposarcoma or smooth muscle sarcoma (81).

#### 4.4.4 Immunotherapy

Immunotherapy for soft tissue sarcomas is a new field that is beginning to demonstrate positive effects. In WDLPS and DDLPS, immune checkpoint inhibitors (ICIs) have also been investigated (82, 83). Expression of PD-1 on tumor infiltration lymphocytes (TILs) and the expression of Programmed Death Ligar-1 (PD-L1) on the cellular membrane of cancer cells may induce an immune reaction which acts as an environmental protection for cancer cells. By inhibition of this interaction, Anti-PD-1 antibodies might overcome tumor immunity resulting in their eventual targeted death (50). PD-1 overexpressing has been found in osteosarcoma, chondrosarcoma, rhabdomyosarcoma, and in liposarcoma variants, while PD - L1 expression is rare (84), therefore, anti-PD-1 antibody can be considered a potential therapeutic option. Pembrolizumab (anti PD-1) has been investigated as a therapeutic option in liposarcoma. The SARC028 study investigated pembrolizumab induced immune checkpoint blockade in soft tissue sarcoma cases (*n* = 40). In 18% of patients with soft tissue sarcoma there was an objective response, whereas in 20% liposarcoma patients there was a partial response, 33% reduction in tumor size, and a median PFS of 25 weeks, while 4 other patients remained in SD, indicating that pembrolizumab was a possible treatment option (85). One study also observed two new checkpoints: pro-apoptotic TIM-3 and anti-proliferative LAG-3, and these new targeted immune checkpoint receptors are under further investigation (86, 87). These results indicate that a thorough study of the nature of cancer micro-environment is necessary in order to develop a better immunotherapeutic approach to combat a variety of sarcomas.

### 4.5 Prognosis

The prognosis of DDLPS is determined by the degree of resection of the tumour, the degree of tumor, the primary condition within and outside the retroperitoneum, and whether there is metastasis (8, 31). A recent NCDB review showed that 5 and 10 years of survival were 51.5% and 34.8% in all patients with primary DDLPS, respectively. However, the corresponding survival probabilities for the subgroup of patients with retroperitoneal or abdominal DDLPS were 42.6% and 25.7%, respectively, which were the lowest for all primary sites. Additional factors that have contributed to a lower OS were the primary tumor size above 10 cm, the higher tumour grade, old patient age and the advanced stage. And the worst prognosis for metastatic disease, with a median survival of 10.2 months (34). Some studies have shown that MDM2/HMGA2 ratio and histologic tumor grading were identified as important prognostic factors, in which more than twice the amplification or gain level of MDM2 than HMGA2 was strongly associated with poor OS (*P* < 0.001) and distant metastasis-free survival (DMFS) (*P* < 0.001), and histologic tumor grading, cellular heterogeneity, and MDM2 immunoreactivity correlated with OS, whereas HMGA2 immunoreactivity tended to correlate with OS. Cellular heterogeneity was also associated with DMFS (88). Myogenic differentiation (MD) has also been recognized as a poor prognostic factor in DDLPS and has a statistically significant effect on DFS and OS. However, the differences are small and may be of little clinical utility (89).

## 5 Conclusion

The patient we described in this report, the tumor protruded locally into the adjacent liver, was poorly demarcated from the liver and duodenum, and had a complex peripheral anatomical location, with no remote metastases at present. The gallbladder as well as the tumor were completely removed During the operation. Following up, she was well physically and mentally, but there was no clear view on the future course of therapy. In conclusion, dedifferentiated liposarcoma is a rare and difficult-to-diagnose tumor. In the early clinical stage, there is no apparent special symptoms of patients, only some patients develop some non-specific symptoms related to tumor compression. For this disease, the imaging manifestations should be summarized, and if the benign or malignant nature of the tumor cannot be defined, a tumor aspiration biopsy should be performed actively. Based on the pathologic findings, it is necessary to carry out radiation therapy during the perioperative period so as to decrease surgical risk and relapse. At present, there is no clear guideline for the treatment of this disease, and chemotherapy and comprehensive treatment still need further in-depth study.

## Data Availability

The original contributions presented in the study are included in the article/Supplementary Material, further inquiries can be directed to the corresponding author.
